# Inadvertent ligation of the left pulmonary artery during intended ductal ligation

**DOI:** 10.1186/s13104-015-1467-3

**Published:** 2015-09-30

**Authors:** Endale Tefera, Ramon Bermudez-Cañete, Carin van Doorn

**Affiliations:** Cardiology Unit, Department of Pediatrics and Child Heath, School of Medicine, Addis Ababa University, Corner of Zambia and T. Abanefso road, P.O.Box 1768, Addis Ababa, Ethiopia; Department of Pediatric Cardiology, Ramon y Cajal University Hospital, Madrid, Spain; Congenital Cardiac Unit, Leeds Teaching Hospital NHS Trust, Leeds, UK

**Keywords:** Inadvertent ligation of left pulmonary artery, Patent ductus arteriosus, Left pulmonary artery reconstruction, Left pulmonary artery stenosis, Left pulmonary artery stenting

## Abstract

**Background:**

Inadvertent ligation of the left pulmonary artery during attempted surgical closure of a Patent Ductus Arteriosus has long been recognized as one of the less common complications of this procedure. Surgical reconstruction of the left pulmonary artery was then often attempted but was difficult or impossible in some of the patients with hypoplasia of the left pulmonary artery and the left lung.

**Case presentation:**

A 10-year-old girl presented with marked exercise intolerance and palpitations and was diagnosed to have large PDA. She had feeding difficulty, diaphoresis, failure to gain weight, recurrent chest infections during infancy and early childhood. Physical examination revealed an underweight child with wide pulse pressure and bounding peripheral pulses. She had active precordium with accentuated P_2_ and machinery murmur in the left 2nd intercostal space and mid diastolic rumble at the mitral area. Echocardiography showed a 12 mm patent arterial duct. She was taken for an intended surgical ligation of the duct but a control echocardiogram on the 3rd postoperative day revealed that the left pulmonary artery, instead of the duct, was ligated. Surgical reconstruction of the left pulmonary artery was undertaken 3 years later, however, this was complicated by post reconstruction left pulmonary artery stenosis. Successful percutaneous stenting of the left pulmonary artery was performed 18 months after the surgical reconstruction.

**Conclusion:**

The incidence of inadvertent LPA ligation may be underestimated where PDA ligation is done by less experienced surgeons and postoperative echocardiography is not routinely performed. Late correction of inadvertent LPA ligation is an important surgical challenge, especially if the duct is still patent. Percutaneous stenting as a primary option may carry significant risk, as the ligated pulmonary artery is fragile. In our case, a good result was achieved with surgical repair followed by percutaneous stenting.

## Background

Inadvertent ligation of the left pulmonary artery (LPA) during attempted surgical closure of a Patent Ductus Arteriosus (PDA) has long been recognized as one of the less common complications of this procedure [1–3]. In a large series by Panagopoulos et al. [4], containing 936 surgical closure of PDA, there was 1 inadvertent left pulmonary artery ligation. In a report from seven different centers, there were four out of ten deaths related to this complication. Recognition of the complication ranged from 1 day to as long as 5 years after the surgery [4]. Surgical reconstruction of the left pulmonary artery was then often attempted but was difficult or impossible in some of the patients with hypoplasia of the left pulmonary artery and the left lung [2]. We report a case of inadvertent ligation of the LPA during attempted PDA closure, and the subsequent reconstruction of the LPA 3 years later, followed by percutaneous stenting for post-surgical LPA stenosis.

## Case presentation

A 10-year-old girl (at the time of first presentation) presented with marked exercise intolerance and palpitations and was diagnosed to have large PDA. She had feeding difficulty, diaphoresis, failure to gain weight, recurrent chest infections during infancy and early childhood. She had no previous hospital admissions or documented episode of infective endocarditis and was not on any cardiac medication.

On physical examination, her weight was 25 kg, and height was 132 cm. Her blood pressure was 125/55 mmHg. She had no pallor or cyanosis. Her peripheral pulses were bounding. Her precordium was active with the point of maximum intensity shifted downwards and laterally. She had thrill over the left second intercostal space. Auscultation revealed accentuated P_2_, continuous murmur over the left 2nd intercostal space and loud diastolic rumble at the mitral area. There was no organomegaly or peripheral edema.

**Fig. 1:**
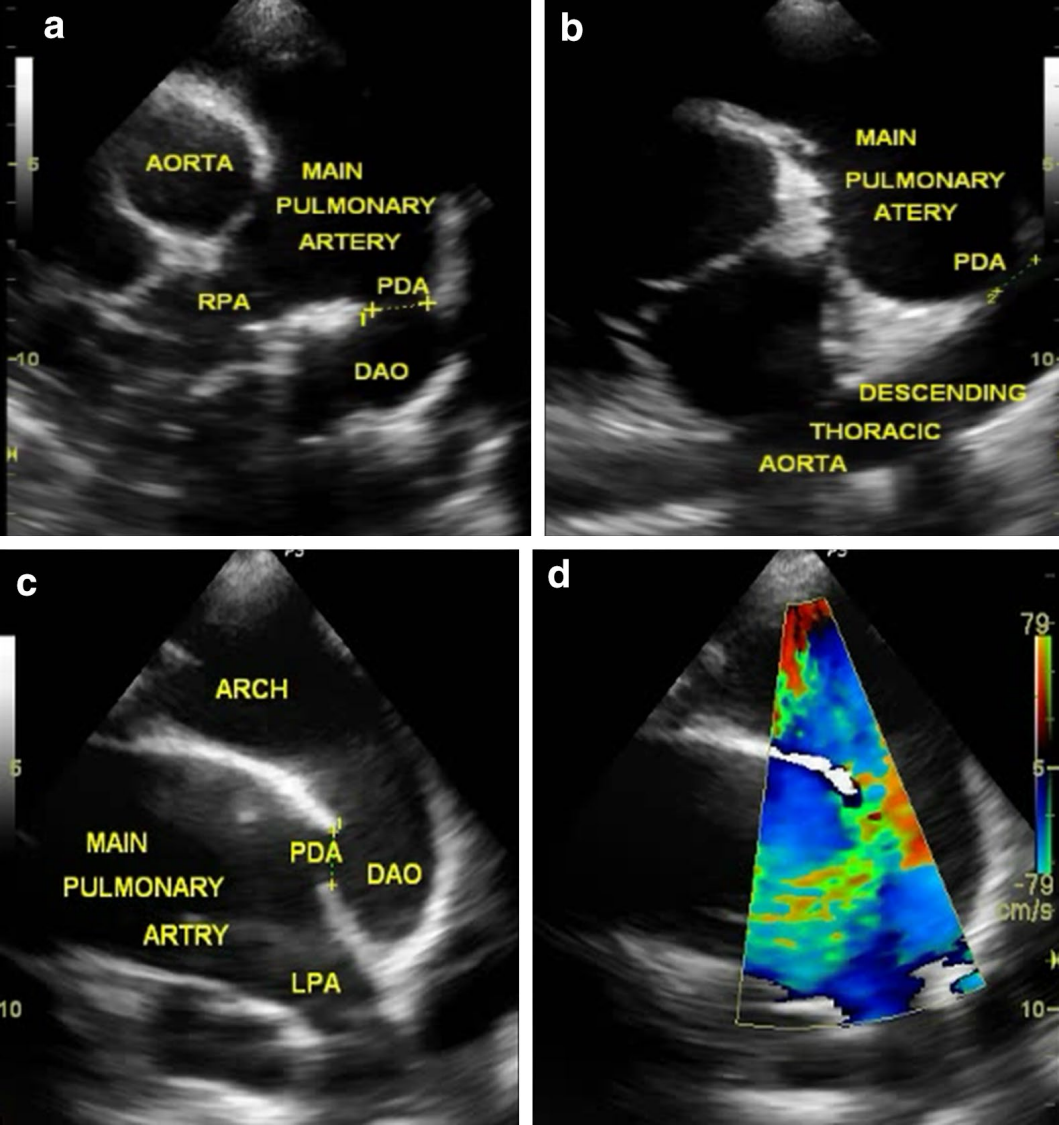
Echocardiographic frames showing large window-type PDA (**a** Parasternal short axis view; **b** Modified duct-cut view; **c** Suprasternal view; **d** Suprasternal view with *color* flow)

Chest X-ray showed cardiomegaly with pulmonary plethora. Electrocardiogram showed left atrial and left ventricular enlargement. On Echocardiography there was a dilated left atrium, left ventricle and pulmonary artery. There was a large window-type PDA measuring about 11–12 mm in diameter (Fig. 1), with left to right shunt at peak systolic velocity of 3.3 m/s. Left ventricular systolic function was normal with an ejection fraction of 61 % and fiber shortening fraction of 32 %. There was minimal mitral regurgitation.

One year after diagnosis, an attempt of percutaneous closure of the PDA was not successful because no suitable size device was available to occlude the expansile duct. Her pulmonary artery pressure was 34/21 mmHg (mean 27 mmHg). She then underwent ductal ligation through a left postero-lateral thoracotomy (mini-thoracotomy) at the local hospital. However, a control echocardiogram done on the third postoperative day showed that the left pulmonary artery instead of the duct was ligated. Consensus was made that removal of the ligature should be done under cardiopulmonary bypass. However, because of lack of facilities and proper cardiac surgical team, it was not possible to do the proposed procedure. So it was decided that she should be on follow up with anticipation of surgical repair during an overseas cardiac surgery missions. The girl was then lost to follow-up, apparently because of clinical improvement of her symptoms.

**Fig. 2:**
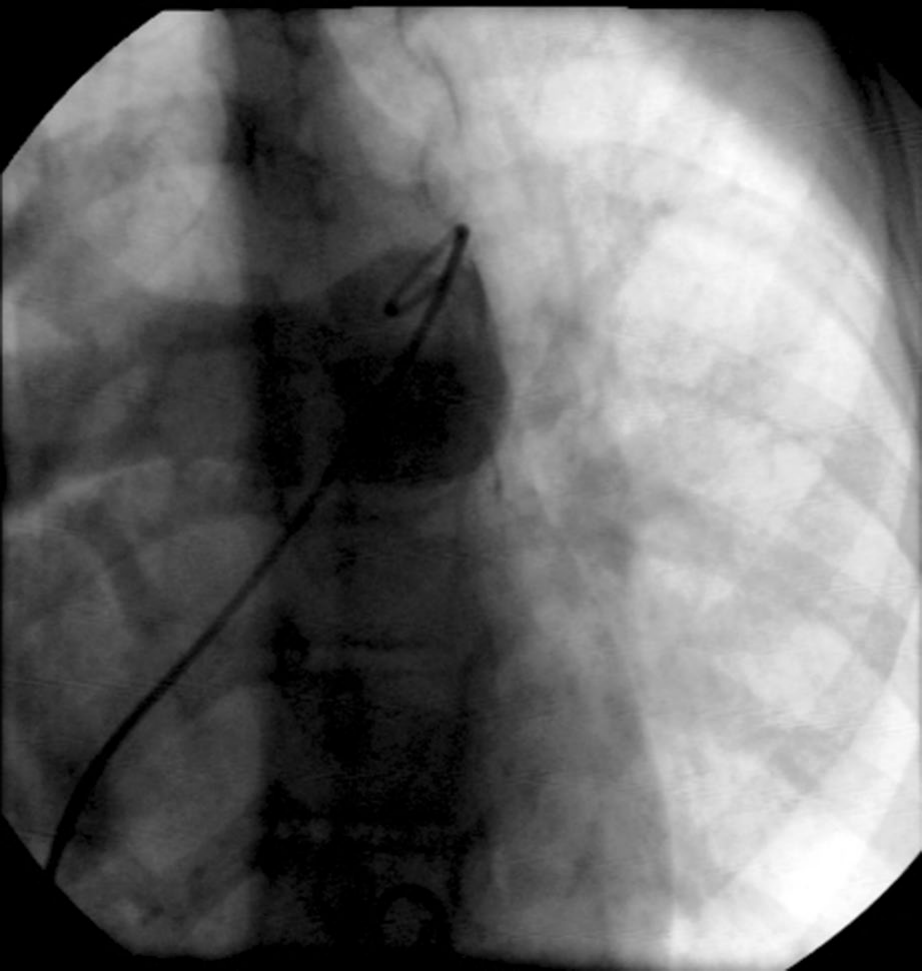
Pulmonary angiogram showing absent LPA and large RPA

**Fig. 3:**
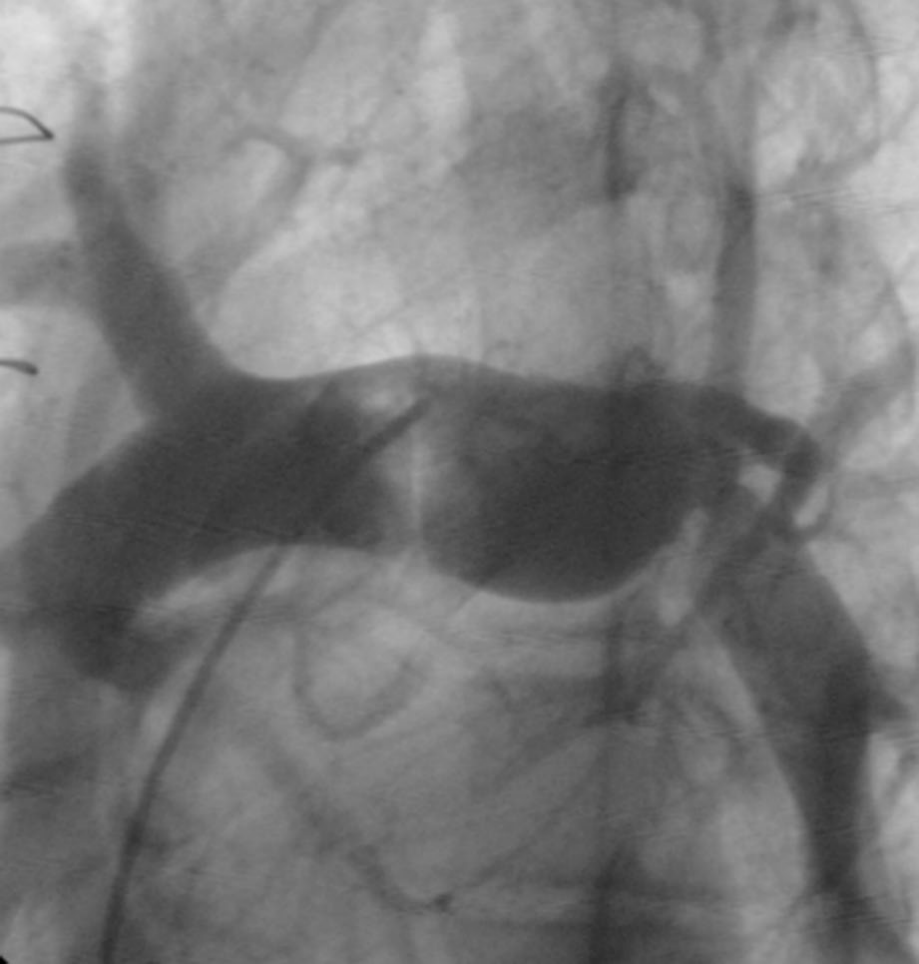
Pulmonary angiogram 6 months after surgical reconstruction, showing discrete LPA stenosis

After 3 years, the girl was traced. She then underwent diagnostic angiogram, which confirmed ligation of the LPA with faint filling of the distal vessel (Fig. 2). Pulmonary artery pressure was 75/49 mmHg (mean 63 mmHg) while aortic pressure was 112/50 mmHg (mean 86 mmHg). During an overseas surgical mission, she subsequently underwent LPA reconstruction and PDA division via median sternotomy and on cardiopulmonary bypass. The LPA had been ligated just proximal to the lobar branching and a small, 3 mm distal orifice was found free from thrombus and with backflow of blood. During removal of the ligature, the fragile LPA wall disintegrated and was reconstructed with difficulty using autologous pericardium. Postoperative recovery was uneventful and she was extubated few hours after transfer to the intensive care unit. Follow-up echocardiogram 3 months later showed significant increment in blood flow through the LPA albeit with residual stenosis. This was confirmed on pulmonary angiogram (Fig. 3).

**Fig. 4:**
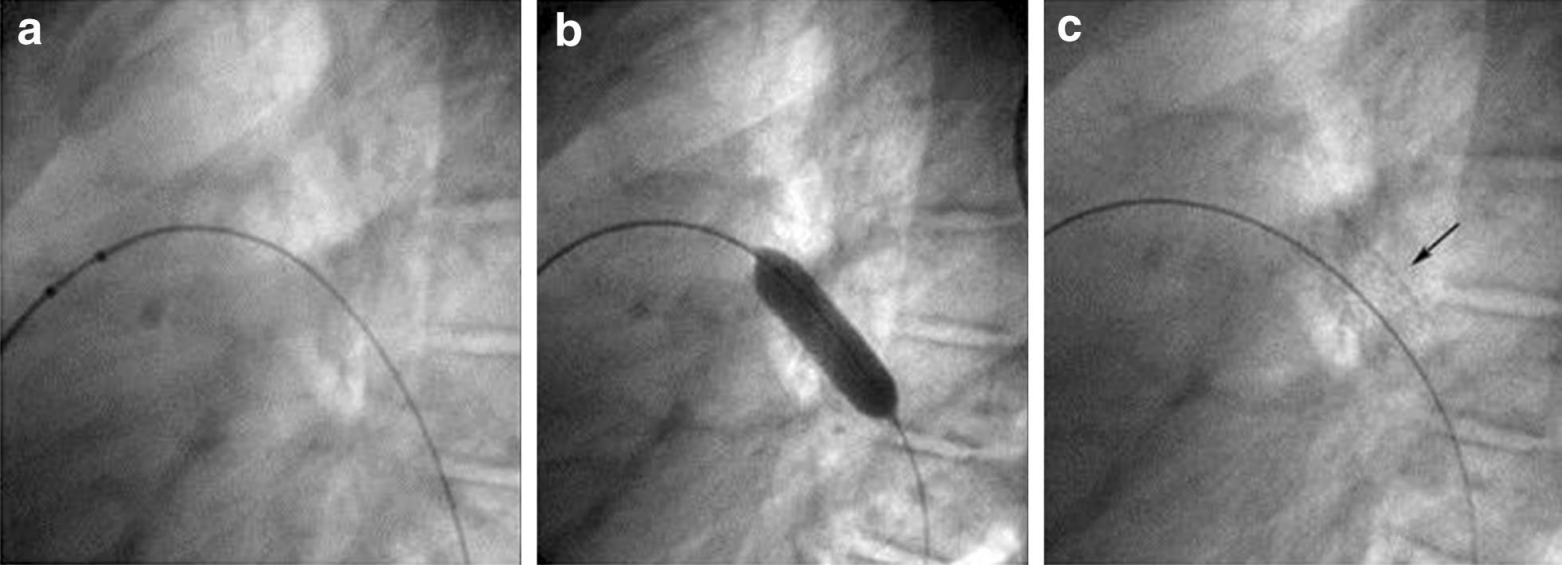
**a** Marked pigtail and super stiff wire in distal LPA; **b** Balloon inflated at 13 *Bars* (stent deployment); **c** Stent in its final position

**Fig. 5:**
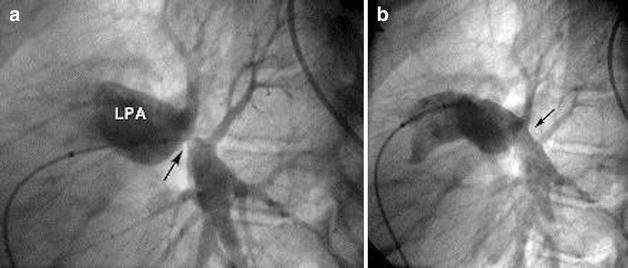
Lateral pulmonary angiogram before stenting (**a**), and after stenting (**b**)

Eighteen months after the surgical reconstruction of the left pulmonary artery, the patient was taken to the catheterization laboratory for a planned percutaneous stenting of the left pulmonary artery. Under general anesthesia, a right femoral vein access was established with an 8F introducer sheath. Intravenous heparin was given at a dose of 100 IU per kilogram of body weight. A 5F Occlu Marker pigtail catheter (pfm medical ag, Wankelstraße 60, Köln, Germany) was advanced over a guide wire and placed in the left pulmonary artery. Angiography was done in straight left lateral projection. There was severe stenosis of the LPA with the stenotic segment measuring 4 mm in diameter and 15 mm in length. An Amplatz superstiff .035″ × 260 cm (AGA Medical, Golden Valley, MN, USA) was advanced deep in the distal LPA. Premounted Valeo^®^ Vascular Stent (C.R. BARD, INC. (GFO) 289 bay road, Queensbury NY, USA), 26 mm in length, crimped over a 10 mm balloon (EV10261C deployment system) was placed across the stenotic segment. The stent was then dilated up to a pressure of 13 bars using a pressure syringe (Fig. 4a–c). Check angiogram showed stable position of the stent. Angiographic comparison of the stenotic segment before and after stenting is shown in Fig. 5. The final diameter of the stent was 10.3 mm. Fluoroscopy time was 8.1 min. There was no any complication during or after the procedure.

## Discussion

The incidence of isolated patent ductus arteriosus (PDA) in term infants is 1 in 2000 live births [5]. It constitutes 6–8 % of all congenital heart defects [6]. Prevalence of patent ductus arteriosus is known to be increased at high altitudes and the effect of altitude is progressive [7, 8]. Furthermore, it has been described that living at high altitude could affect the morphology and size of the duct with angiographic type A (Toronto classification) being more common at high altitudes [8].

The capital city of Ethiopia, Addis Ababa and its surrounding area are located at an altitude of 2100–2800 meters above sea level. PDAs are common and Ligation of the patent duct via left thoracotomy used to be the treatment of choice. However, this is increasingly replaced by percutaneous device closure, [9, 10]. The incidence of inadvertent LPA ligation is estimated to be almost about 1 in 1000 cases [4]. However, this is probably an underestimate considering the lack of routine echocardiography facilities in many centers in developing countries.

In our Institution, we started to recall patients with PDA ligation for follow up echo in 2009 after it was recognized that some inadvertent LPA ligations had taken place. Seven out of close to 100 PDA ligation cases were found to have inadvertent ligation of the left pulmonary artery. With increased awareness of this complication by both cardiology and surgical teams, no further inadvertent LPA ligations have been identified over the last 6 years.

Inadvertent LPA ligation can be avoided with good surgical discipline. After left postero-lateral thoracotomy, the lung is retracted anteriorly. The PDA, together with the distal aortic arch and proximal descending aorta should be identified, as well as the recurrent laryngeal nerve, which courses around the PDA. Only then should PDA ligation be performed. A common mistake is to retract the lung downwards at the time of surgery, and mistake the LPA for the duct. Follow up echo should always be performed after PDA ligation, ideally in the immediate postoperative period. At this time, it is still possible to take the patient back to the operating theatre for repeat thoracotomy, PDA ligation and removal of ligature around LPA. However, the procedure carries a significant risk of LPA injury.

Late correction of inadvertent LPA ligation is an important surgical challenge, especially if the duct is still patent. We performed the procedure via median sternotomy and on cardiopulmonary bypass. Early during the operation, immediately after bypass has been established, the heart was kept loaded to maintain ejection and avoid run off the arterial bypass flow into the pulmonary vascular bed. With the heart beating, the patient is cooled to 28C and during a very short period of circulatory arrest, the PDA is controlled. After restarting the circulation, The LPA was subsequently repaired during a short period of cardioplegic arrest. In the beginning, we feared the distal LPA could be thrombosed, however pre-operative angiography demonstrated a patent distal left pulmonary artery on venous wedge injection. Surgical repair was complicated by the fragile tissues resulting in residual stenosis, which was later corrected by stenting.

## Conclusion

The incidence of inadvertent LPA ligation may be underestimated where PDA ligation is done by less experienced surgeons and postoperative echocardiography is not routinely performed. We are reporting this case to emphasize the need for routine postoperative echocardiography following surgical closure of PDA, especially if mini-thoracotomy is used. Late correction of inadvertent LPA ligation is an important surgical challenge, especially if the duct is still patent. Percutaneous stenting as a primary option may carry significant risk, as the ligated pulmonary artery is fragile. In our case, a good anatomical result was achieved with a combination of surgery and intervention.

## Consent

Written informed consent was obtained from the patient’s parents for publication of this Case report and any accompanying images. A copy of the written consent is available for review by the Editor of this journal.

## Abbreviations

LPA: left pulmonary artery
PA: pulmonary artery
PDA: patent ductus arteriosus
